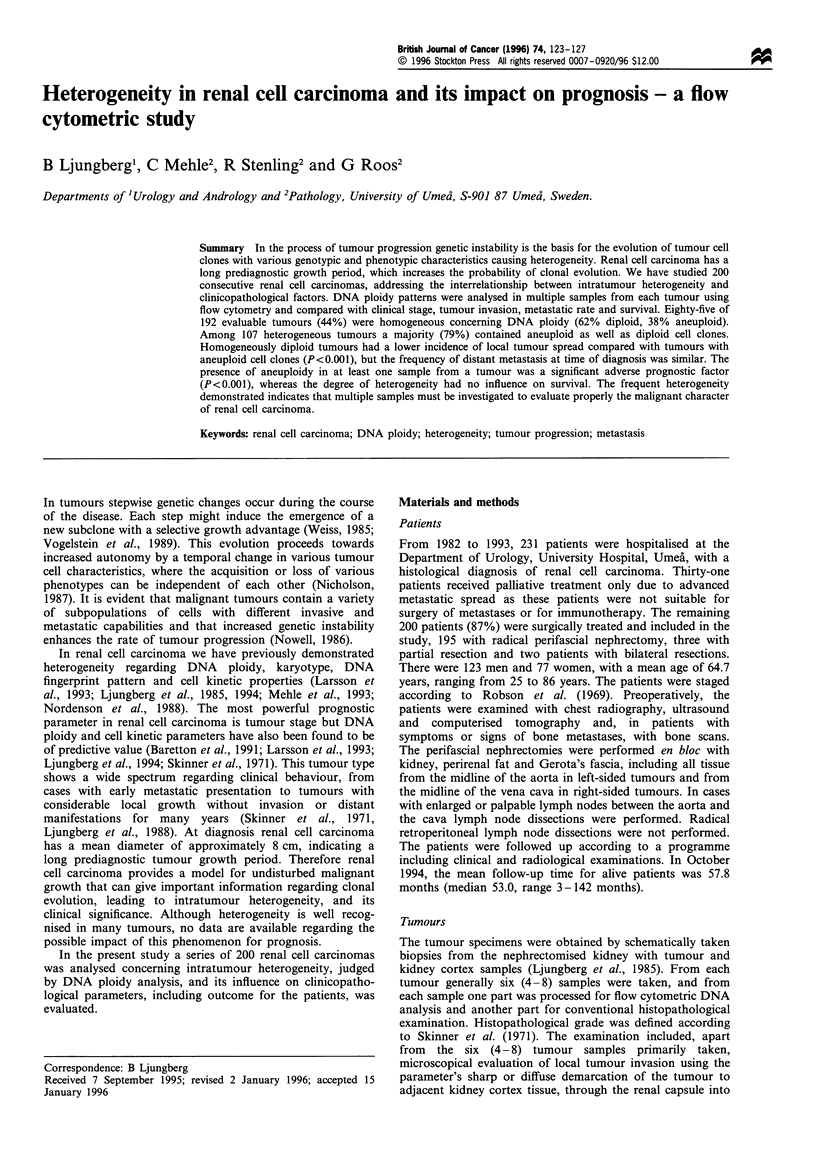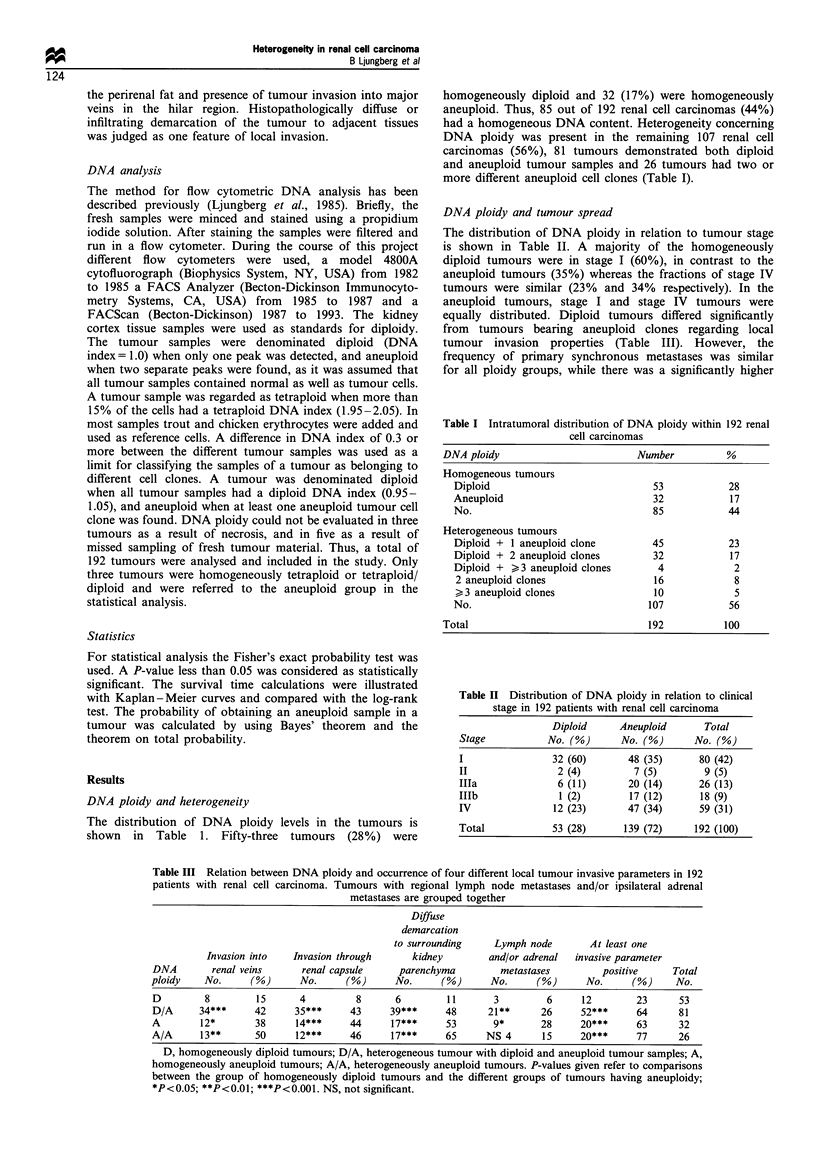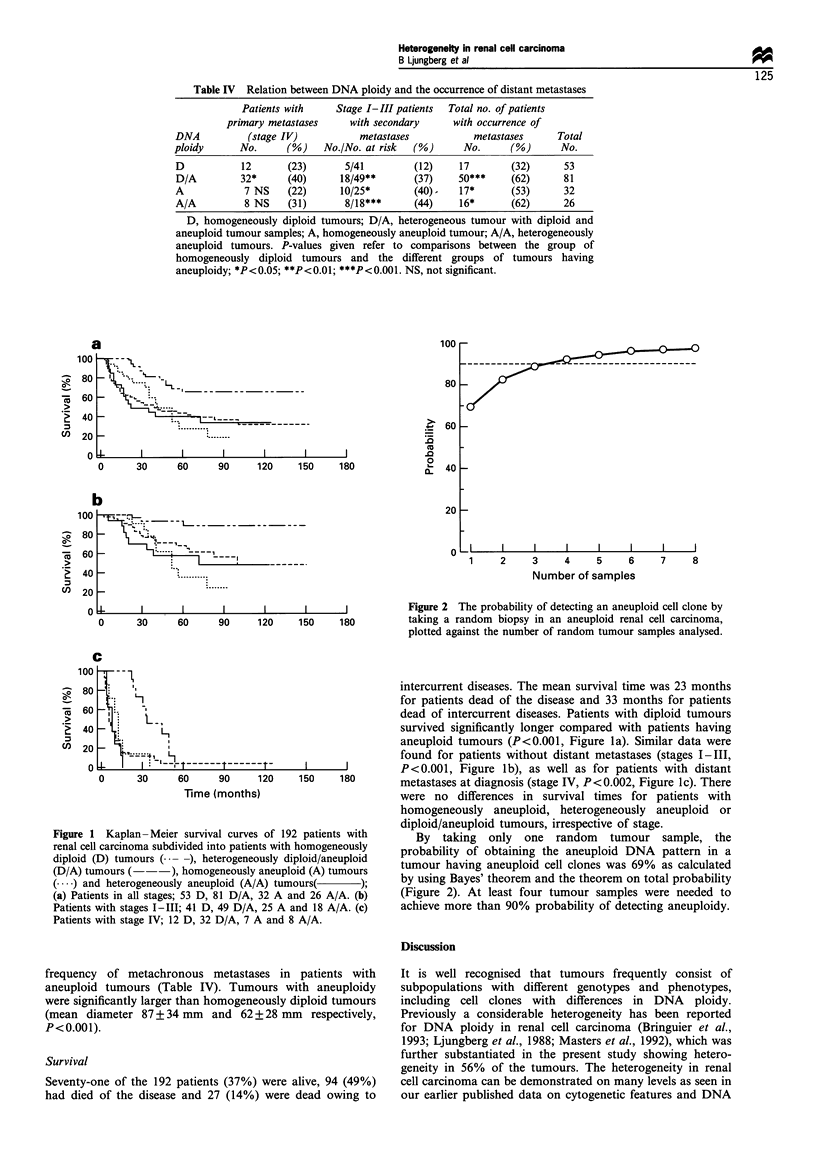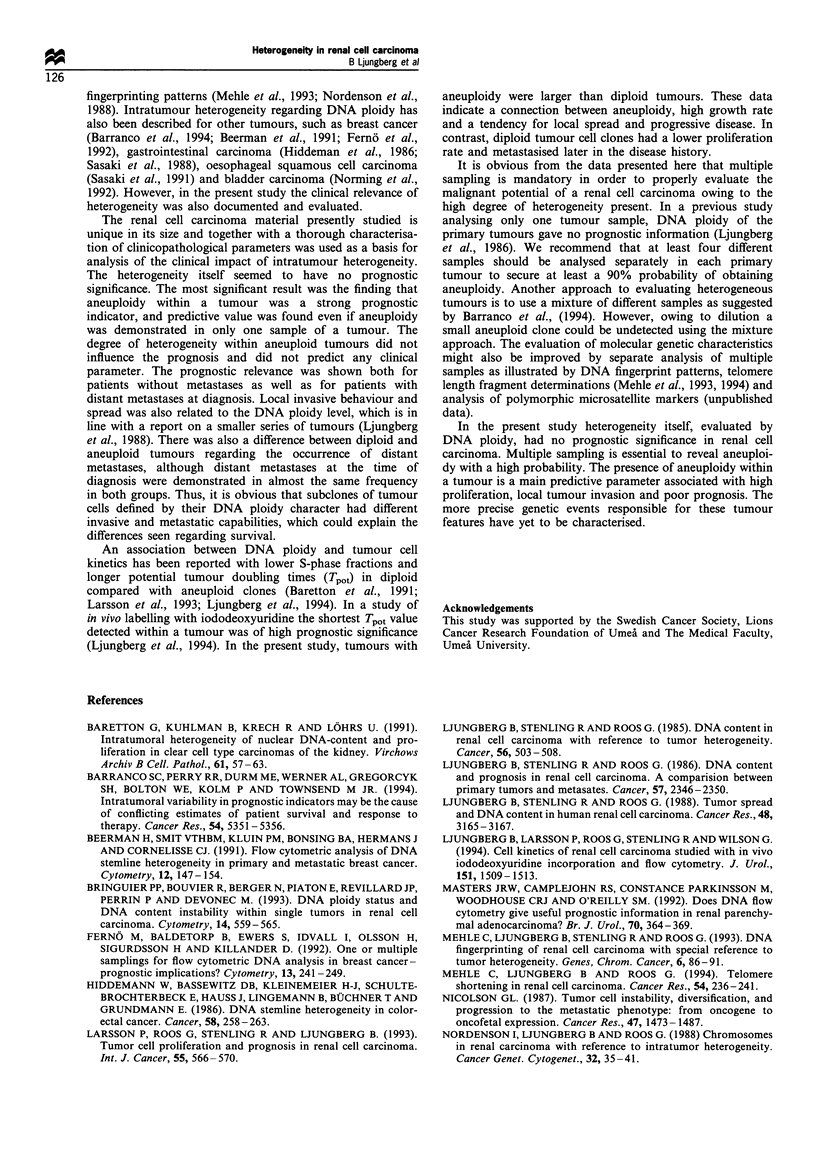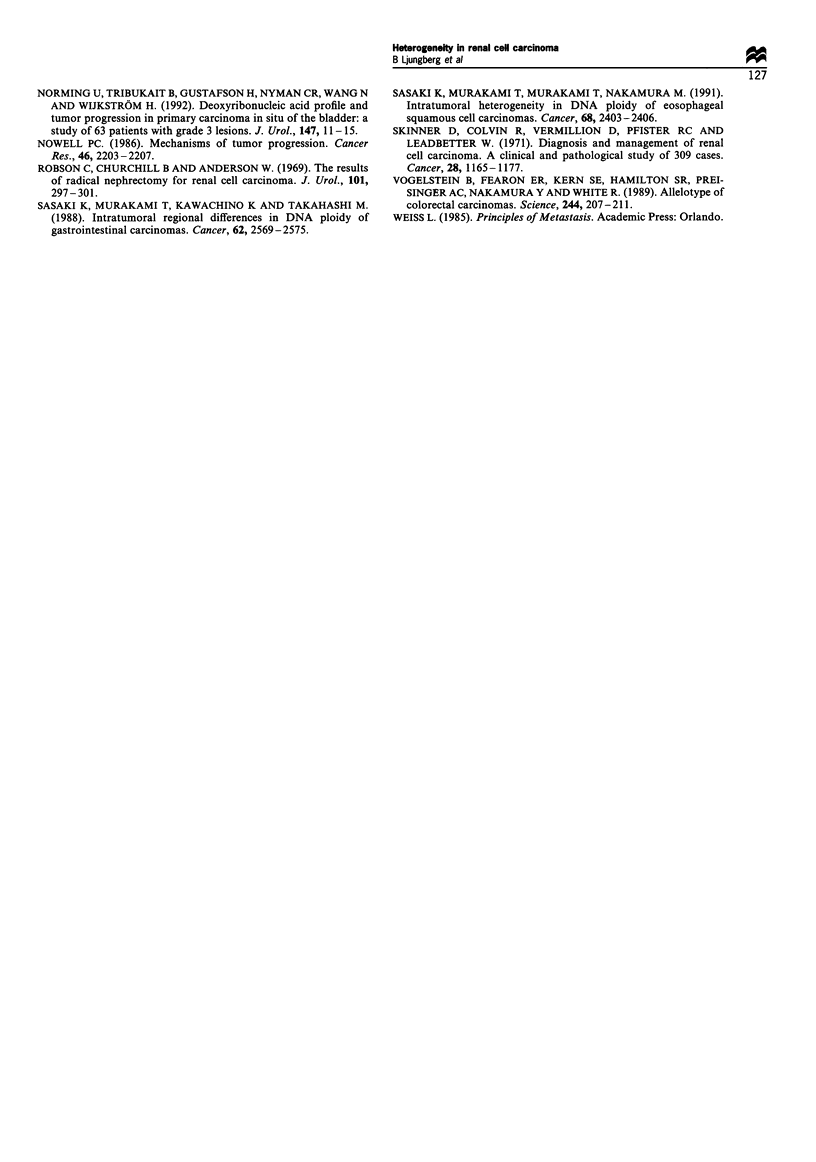# Heterogeneity in renal cell carcinoma and its impact no prognosis--a flow cytometric study.

**DOI:** 10.1038/bjc.1996.326

**Published:** 1996-07

**Authors:** B. Ljungberg, C. Mehle, R. Stenling, G. Roos

**Affiliations:** Department of Urology and Andrology, University of Umeå, Sweden.

## Abstract

In the process of tumour progression genetic instability is the basis for the evolution of tumour cell clones with various genotypic and phenotypic characteristics causing heterogeneity. Renal cell carcinoma has a long prediagnostic growth period, which increases the probability of clonal evolution. We have studied 200 consecutive renal cell carcinomas, addressing the interrelationship between intratumour heterogeneity and clinicopathological factors. DNA ploidy patterns were analysed in multiple samples from each tumour using flow cytometry and compared with clinical stage, tumour invasion, metastatic rate and survival. Eighty-five of 192 evaluable tumours (44%) were homogeneous concerning DNA ploidy (62% diploid, 38% aneuploid). Among 107 heterogeneous tumours a majority (79%) contained aneuploid as well as diploid cell clones. Homogeneously diploid tumours had a lower incidence of local tumour spread compared with tumours with aneuploid cell clones (P < or = 0.001), but the frequency of distant metastasis at time of diagnosis was similar. The presence of aneuploidy in at least one sample from a tumour was a significant adverse prognostic factor (P < 0.001), whereas the degree of heterogeneity had no influence on survival. The frequent heterogeneity demonstrated indicates that multiple samples must be investigated to evaluate properly the malignant character of renal cell carcinoma.


British Journal of Cancer (1996) 74, 123-127
© 1996 Stockton Press All rights reserved 0007-0920/96 $12.00

### Heterogeneity in renal cell carcinoma and its impact on prognosis – a flow cytometric study

B Ljungberg[1], C Mehle[2], R Stenling[2] and G Roos[2]

*Departments of [1]Urology and Andrology and [2]Pathology, University of Umeå, S-901 87 Umeå, Sweden.*

**Summary** In the process of tumour progression genetic instability is the basis for the evolution of tumour cell clones with various genotypic and phenotypic characteristics causing heterogeneity. Renal cell carcinoma has a long prediagnostic growth period, which increases the probability of clonal evolution. We have studied 200 consecutive renal cell carcinomas, addressing the interrelationship between intratumour heterogeneity and clinicopathological factors. DNA ploidy patterns were analysed in multiple samples from each tumour using flow cytometry and compared with clinical stage, tumour invasion, metastatic rate and survival. Eighty-five of 192 evaluable tumours (44%) were homogeneous concerning DNA ploidy (62% diploid, 38% aneuploid). Among 107 heterogeneous tumours a majority (79%) contained aneuploid as well as diploid cell clones. Homogeneously diploid tumours had a lower incidence of local tumour spread compared with tumours with aneuploid cell clones ($P < 0.001$), but the frequency of distant metastasis at time of diagnosis was similar. The presence of aneuploidy in at least one sample from a tumour was a significant adverse prognostic factor ($P < 0.001$), whereas the degree of heterogeneity had no influence on survival. The frequent heterogeneity demonstrated indicates that multiple samples must be investigated to evaluate properly the malignant character of renal cell carcinoma.

**Keywords:** renal cell carcinoma; DNA ploidy; heterogeneity; tumour progression; metastasis

In tumours stepwise genetic changes occur during the course of the disease. Each step might induce the emergence of a new subclone with a selective growth advantage (Weiss, 1985; Vogelstein *et al.*, 1989). This evolution proceeds towards increased autonomy by a temporal change in various tumour cell characteristics, where the acquisition or loss of various phenotypes can be independent of each other (Nicholson, 1987). It is evident that malignant tumours contain a variety of subpopulations of cells with different invasive and metastatic capabilities and that increased genetic instability enhances the rate of tumour progression (Nowell, 1986).

In renal cell carcinoma we have previously demonstrated heterogeneity regarding DNA ploidy, karyotype, DNA fingerprint pattern and cell kinetic properties (Larsson *et al.*, 1993; Ljungberg *et al.*, 1985, 1994; Mehle *et al.*, 1993; Nordenson *et al.*, 1988). The most powerful prognostic parameter in renal cell carcinoma is tumour stage but DNA ploidy and cell kinetic parameters have also been found to be of predictive value (Baretton *et al.*, 1991; Larsson *et al.*, 1993; Ljungberg *et al.*, 1994; Skinner *et al.*, 1971). This tumour type shows a wide spectrum regarding clinical behaviour, from cases with early metastatic presentation to tumours with considerable local growth without invasion or distant manifestations for many years (Skinner *et al.*, 1971, Ljungberg *et al.*, 1988). At diagnosis renal cell carcinoma has a mean diameter of approximately 8 cm, indicating a long prediagnostic tumour growth period. Therefore renal cell carcinoma provides a model for undisturbed malignant growth that can give important information regarding clonal evolution, leading to intratumour heterogeneity, and its clinical significance. Although heterogeneity is well recognised in many tumours, no data are available regarding the possible impact of this phenomenon for prognosis.

In the present study a series of 200 renal cell carcinomas was analysed concerning intratumour heterogeneity, judged by DNA ploidy analysis, and its influence on clinicopathological parameters, including outcome for the patients, was evaluated.

Correspondence: B Ljungberg
Received 7 September 1995; revised 2 January 1996; accepted 15 January 1996

### Materials and methods

### *Patients*

From 1982 to 1993, 231 patients were hospitalised at the Department of Urology, University Hospital, Umeå, with a histological diagnosis of renal cell carcinoma. Thirty-one patients received palliative treatment only due to advanced metastatic spread as these patients were not suitable for surgery of metastases or for immunotherapy. The remaining 200 patients (87%) were surgically treated and included in the study, 195 with radical perifascial nephrectomy, three with partial resection and two patients with bilateral resections. There were 123 men and 77 women, with a mean age of 64.7 years, ranging from 25 to 86 years. The patients were staged according to Robson *et al.* (1969). Preoperatively, the patients were examined with chest radiography, ultrasound and computerised tomography and, in patients with symptoms or signs of bone metastases, with bone scans. The perifascial nephrectomies were performed *en bloc* with kidney, perirenal fat and Gerota's fascia, including all tissue from the midline of the aorta in left-sided tumours and from the midline of the vena cava in right-sided tumours. In cases with enlarged or palpable lymph nodes between the aorta and the cava lymph node dissections were performed. Radical retroperitoneal lymph node dissections were not performed. The patients were followed up according to a programme including clinical and radiological examinations. In October 1994, the mean follow-up time for alive patients was 57.8 months (median 53.0, range 3-142 months).

### *Tumours*

The tumour specimens were obtained by schematically taken biopsies from the nephrectomised kidney with tumour and kidney cortex samples (Ljungberg *et al.*, 1985). From each tumour generally six (4-8) samples were taken, and from each sample one part was processed for flow cytometric DNA analysis and another part for conventional histopathological examination. Histopathological grade was defined according to Skinner *et al.* (1971). The examination included, apart from the six (4-8) tumour samples primarily taken, microscopical evaluation of local tumour invasion using the parameter's sharp or diffuse demarcation of the tumour to adjacent kidney cortex tissue, through the renal capsule into the perirenal fat and presence of tumour invasion into major veins in the hilar region. Histopathologically diffuse or infiltrating demarcation of the tumour to adjacent tissues was judged as one feature of local invasion.

### *DNA analysis*

The method for flow cytometric DNA analysis has been described previously (Ljungberg *et al.*, 1985). Briefly, the fresh samples were minced and stained using a propidium iodide solution. After staining the samples were filtered and run in a flow cytometer. During the course of this project different flow cytometers were used, a model 4800A cytofluorograph (Biophysics System, NY, USA) from 1982 to 1985 a FACS Analyzer (Becton-Dickinson Immunocytometry Systems, CA, USA) from 1985 to 1987 and a FACScan (Becton-Dickinson) 1987 to 1993. The kidney cortex tissue samples were used as standards for diploidy. The tumour samples were denominated diploid (DNA index = 1.0) when only one peak was detected, and aneuploid when two separate peaks were found, as it was assumed that all tumour samples contained normal as well as tumour cells. A tumour sample was regarded as tetraploid when more than 15% of the cells had a tetraploid DNA index (1.95–2.05). In most samples trout and chicken erythrocytes were added and used as reference cells. A difference in DNA index of 0.3 or more between the different tumour samples was used as a limit for classifying the samples of a tumour as belonging to different cell clones. A tumour was denominated diploid when all tumour samples had a diploid DNA index (0.95–1.05), and aneuploid when at least one aneuploid tumour cell clone was found. DNA ploidy could not be evaluated in three tumours as a result of necrosis, and in five as a result of missed sampling of fresh tumour material. Thus, a total of 192 tumours were analysed and included in the study. Only three tumours were homogeneously tetraploid or tetraploid/diploid and were referred to the aneuploid group in the statistical analysis.

### *Statistics*

For statistical analysis the Fisher's exact probability test was used. A $P$-value less than 0.05 was considered as statistically significant. The survival time calculations were illustrated with Kaplan–Meier curves and compared with the log-rank test. The probability of obtaining an aneuploid sample in a tumour was calculated by using Bayes' theorem and the theorem on total probability.

### Results

### *DNA ploidy and heterogeneity*

The distribution of DNA ploidy levels in the tumours is shown in Table 1. Fifty-three tumours (28%) were homogeneously diploid and 32 (17%) were homogeneously aneuploid. Thus, 85 out of 192 renal cell carcinomas (44%) had a homogeneous DNA content. Heterogeneity concerning DNA ploidy was present in the remaining 107 renal cell carcinomas (56%), 81 tumours demonstrated both diploid and aneuploid tumour samples and 26 tumours had two or more different aneuploid cell clones (Table I).

### *DNA ploidy and tumour spread*

The distribution of DNA ploidy in relation to tumour stage is shown in Table II. A majority of the homogeneously diploid tumours were in stage I (60%), in contrast to the aneuploid tumours (35%) whereas the fractions of stage IV tumours were similar (23% and 34% respectively). In the aneuploid tumours, stage I and stage IV tumours were equally distributed. Diploid tumours differed significantly from tumours bearing aneuploid clones regarding local tumour invasion properties (Table III). However, the frequency of primary synchronous metastases was similar for all ploidy groups, while there was a significantly higher

**Table I** Intratumoral distribution of DNA ploidy within 192 renal cell carcinomas

| *DNA ploidy* | *Number* | *%* |
|---|---|---|
| Homogeneous tumours | | |
| Diploid | 53 | 28 |
| Aneuploid | 32 | 17 |
| No. | 85 | 44 |
| Heterogeneous tumours | | |
| Diploid + 1 aneuploid clone | 45 | 23 |
| Diploid + 2 aneuploid clones | 32 | 17 |
| Diploid + ⩾3 aneuploid clones | 4 | 2 |
| 2 aneuploid clones | 16 | 8 |
| ⩾3 aneuploid clones | 10 | 5 |
| No. | 107 | 56 |
| Total | 192 | 100 |

**Table II** Distribution of DNA ploidy in relation to clinical stage in 192 patients with renal cell carcinoma

| *Stage* | *Diploid* No. (%) | *Aneuploid* No. (%) | *Total* No. (%) |
|---|---|---|---|
| I | 32 (60) | 48 (35) | 80 (42) |
| II | 2 (4) | 7 (5) | 9 (5) |
| IIIa | 6 (11) | 20 (14) | 26 (13) |
| IIIb | 1 (2) | 17 (12) | 18 (9) |
| IV | 12 (23) | 47 (34) | 59 (31) |
| Total | 53 (28) | 139 (72) | 192 (100) |

**Table III** Relation between DNA ploidy and occurrence of four different local tumour invasive parameters in 192 patients with renal cell carcinoma. Tumours with regional lymph node metastases and/or ipsilateral adrenal metastases are grouped together

| *DNA ploidy* | *Invasion into renal veins* No. | (%) | *Invasion through renal capsule* No. | (%) | *Diffuse demarcation to surrounding kidney parenchyma* No. | (%) | *Lymph node and/or adrenal metastases* No. | (%) | *At least one invasive parameter positive* No. | (%) | *Total* No. |
|---|---|---|---|---|---|---|---|---|---|---|---|
| D | 8 | 15 | 4 | 8 | 6 | 11 | 3 | 6 | 12 | 23 | 53 |
| D/A | 34*** | 42 | 35*** | 43 | 39*** | 48 | 21** | 26 | 52*** | 64 | 81 |
| A | 12* | 38 | 14*** | 44 | 17*** | 53 | 9* | 28 | 20*** | 63 | 32 |
| A/A | 13** | 50 | 12*** | 46 | 17*** | 65 | NS 4 | 15 | 20*** | 77 | 26 |

D, homogeneously diploid tumours; D/A, heterogeneous tumour with diploid and aneuploid tumour samples; A, homogeneously aneuploid tumours; A/A, heterogeneously aneuploid tumours. $P$-values given refer to comparisons between the group of homogeneously diploid tumours and the different groups of tumours having aneuploidy; $^{*}P<0.05$; $^{**}P<0.01$; $^{***}P<0.001$. NS, not significant.

**Table IV** Relation between DNA ploidy and the occurrence of distant metastases

| *DNA ploidy* | *Patients with primary metastases (stage IV)* No. | (%) | *Stage I–III patients with secondary metastases* No./No. at risk | (%) | *Total no. of patients with occurrence of metastases* No. | (%) | *Total* No. |
|---|---|---|---|---|---|---|---|
| D | 12 | (23) | 5/41 | (12) | 17 | (32) | 53 |
| D/A | 32* | (40) | 18/49** | (37) | 50*** | (62) | 81 |
| A | 7 NS | (22) | 10/25* | (40) | 17* | (53) | 32 |
| A/A | 8 NS | (31) | 8/18*** | (44) | 16* | (62) | 26 |

D, homogeneously diploid tumours; D/A, heterogeneous tumour with diploid and aneuploid tumour samples; A, homogeneously aneuploid tumour; A/A, heterogeneously aneuploid tumours. *P*-values given refer to comparisons between the group of homogeneously diploid tumours and the different groups of tumours having aneuploidy; *$P<0.05$; **$P<0.01$; ***$P<0.001$. NS, not significant.

**Figure 1** Kaplan–Meier survival curves of 192 patients with renal cell carcinoma subdivided into patients with homogeneously diploid (D) tumours (·-- -), heterogeneously diploid/aneuploid (D/A) tumours (———), homogeneously aneuploid (A) tumours (····) and heterogeneously aneuploid (A/A) tumours(————); (**a**) Patients in all stages; 53 D, 81 D/A, 32 A and 26 A/A. (**b**) Patients with stages I–III; 41 D, 49 D/A, 25 A and 18 A/A. (**c**) Patients with stage IV; 12 D, 32 D/A, 7 A and 8 A/A.

**Figure 2** The probability of detecting an aneuploid cell clone by taking a random biopsy in an aneuploid renal cell carcinoma, plotted against the number of random tumour samples analysed.

frequency of metachronous metastases in patients with aneuploid tumours (Table IV). Tumours with aneuploidy were significantly larger than homogeneously diploid tumours (mean diameter $87\pm34$ mm and $62\pm28$ mm respectively, $P<0.001$).

### *Survival*

Seventy-one of the 192 patients (37%) were alive, 94 (49%) had died of the disease and 27 (14%) were dead owing to intercurrent diseases. The mean survival time was 23 months for patients dead of the disease and 33 months for patients dead of intercurrent diseases. Patients with diploid tumours survived significantly longer compared with patients having aneuploid tumours ($P<0.001$, Figure 1a). Similar data were found for patients without distant metastases (stages I–III, $P<0.001$, Figure 1b), as well as for patients with distant metastases at diagnosis (stage IV, $P<0.002$, Figure 1c). There were no differences in survival times for patients with homogeneously aneuploid, heterogeneously aneuploid or diploid/aneuploid tumours, irrespective of stage.

By taking only one random tumour sample, the probability of obtaining the aneuploid DNA pattern in a tumour having aneuploid cell clones was 69% as calculated by using Bayes' theorem and the theorem on total probability (Figure 2). At least four tumour samples were needed to achieve more than 90% probability of detecting aneuploidy.

### Discussion

It is well recognised that tumours frequently consist of subpopulations with different genotypes and phenotypes, including cell clones with differences in DNA ploidy. Previously a considerable heterogeneity has been reported for DNA ploidy in renal cell carcinoma (Bringuier *et al.*, 1993; Ljungberg *et al.*, 1988; Masters *et al.*, 1992), which was further substantiated in the present study showing heterogeneity in 56% of the tumours. The heterogeneity in renal cell carcinoma can be demonstrated on many levels as seen in our earlier published data on cytogenetic features and DNA

fingerprinting patterns (Mehle *et al.*, 1993; Nordenson *et al.*, 1988). Intratumour heterogeneity regarding DNA ploidy has also been described for other tumours, such as breast cancer (Barranco *et al.*, 1994; Beerman *et al.*, 1991; Fernö *et al.*, 1992), gastrointestinal carcinoma (Hiddeman *et al.*, 1986; Sasaki *et al.*, 1988), oesophageal squamous cell carcinoma (Sasaki *et al.*, 1991) and bladder carcinoma (Norming *et al.*, 1992). However, in the present study the clinical relevance of heterogeneity was also documented and evaluated.

The renal cell carcinoma material presently studied is unique in its size and together with a thorough characterisation of clinicopathological parameters was used as a basis for analysis of the clinical impact of intratumour heterogeneity. The heterogeneity itself seemed to have no prognostic significance. The most significant result was the finding that aneuploidy within a tumour was a strong prognostic indicator, and predictive value was found even if aneuploidy was demonstrated in only one sample of a tumour. The degree of heterogeneity within aneuploid tumours did not influence the prognosis and did not predict any clinical parameter. The prognostic relevance was shown both for patients without metastases as well as for patients with distant metastases at diagnosis. Local invasive behaviour and spread was also related to the DNA ploidy level, which is in line with a report on a smaller series of tumours (Ljungberg *et al.*, 1988). There was also a difference between diploid and aneuploid tumours regarding the occurrence of distant metastases, although distant metastases at the time of diagnosis were demonstrated in almost the same frequency in both groups. Thus, it is obvious that subclones of tumour cells defined by their DNA ploidy character had different invasive and metastatic capabilities, which could explain the differences seen regarding survival.

An association between DNA ploidy and tumour cell kinetics has been reported with lower S-phase fractions and longer potential tumour doubling times ($T_{pot}$) in diploid compared with aneuploid clones (Baretton *et al.*, 1991; Larsson *et al.*, 1993; Ljungberg *et al.*, 1994). In a study of *in vivo* labelling with iododeoxyuridine the shortest $T_{pot}$ value detected within a tumour was of high prognostic significance (Ljungberg *et al.*, 1994). In the present study, tumours with aneuploidy were larger than diploid tumours. These data indicate a connection between aneuploidy, high growth rate and a tendency for local spread and progressive disease. In contrast, diploid tumour cell clones had a lower proliferation rate and metastasised later in the disease history.

It is obvious from the data presented here that multiple sampling is mandatory in order to properly evaluate the malignant potential of a renal cell carcinoma owing to the high degree of heterogeneity present. In a previous study analysing only one tumour sample, DNA ploidy of the primary tumours gave no prognostic information (Ljungberg *et al.*, 1986). We recommend that at least four different samples should be analysed separately in each primary tumour to secure at least a 90% probability of obtaining aneuploidy. Another approach to evaluating heterogeneous tumours is to use a mixture of different samples as suggested by Barranco *et al.*, (1994). However, owing to dilution a small aneuploid clone could be undetected using the mixture approach. The evaluation of molecular genetic characteristics might also be improved by separate analysis of multiple samples as illustrated by DNA fingerprint patterns, telomere length fragment determinations (Mehle *et al.*, 1993, 1994) and analysis of polymorphic microsatellite markers (unpublished data).

In the present study heterogeneity itself, evaluated by DNA ploidy, had no prognostic significance in renal cell carcinoma. Multiple sampling is essential to reveal aneuploidy with a high probability. The presence of aneuploidy within a tumour is a main predictive parameter associated with high proliferation, local tumour invasion and poor prognosis. The more precise genetic events responsible for these tumour features have yet to be characterised.

**Acknowledgements**
This study was supported by the Swedish Cancer Society, Lions Cancer Research Foundation of Umeå and The Medical Faculty, Umeå University.

### References

BARETTON G, KUHLMAN B, KRECH R AND LÖHRS U. (1991). Intratumoral heterogeneity of nuclear DNA-content and proliferation in clear cell type carcinomas of the kidney. *Virchows Archiv B Cell. Pathol.*, **61**, 57–63.

BARRANCO SC, PERRY RR, DURM ME, WERNER AL, GREGORCYK SH, BOLTON WE, KOLM P AND TOWNSEND M JR. (1994). Intratumoral variability in prognostic indicators may be the cause of conflicting estimates of patient survival and response to therapy. *Cancer Res.*, **54**, 5351–5356.

BEERMAN H, SMIT VTHBM, KLUIN PM, BONSING BA, HERMANS J AND CORNELISSE CJ. (1991). Flow cytometric analysis of DNA stemline heterogeneity in primary and metastatic breast cancer. *Cytometry*, **12**, 147–154.

BRINGUIER PP, BOUVIER R, BERGER N, PIATON E, REVILLARD JP, PERRIN P AND DEVONEC M. (1993). DNA ploidy status and DNA content instability within single tumors in renal cell carcinoma. *Cytometry*, **14**, 559–565.

FERNÖ M, BALDETORP B, EWERS S, IDVALL I, OLSSON H, SIGURDSSON H AND KILLANDER D. (1992). One or multiple samplings for flow cytometric DNA analysis in breast cancer – prognostic implications? *Cytometry*, **13**, 241–249.

HIDDEMANN W, BASSEWITZ DB, KLEINEMEIER H-J, SCHULTE-BROCHTERBECK E, HAUSS J, LINGEMANN B, BÜCHNER T AND GRUNDMANN E. (1986). DNA stemline heterogeneity in colorectal cancer. *Cancer*, **58**, 258–263.

LARSSON P, ROOS G, STENLING R AND LJUNGBERG B. (1993). Tumor cell proliferation and prognosis in renal cell carcinoma. *Int. J. Cancer*, **55**, 566–570.

LJUNGBERG B, STENLING R AND ROOS G. (1985). DNA content in renal cell carcinoma with reference to tumor heterogeneity. *Cancer*, **56**, 503–508.

LJUNGBERG B, STENLING R AND ROOS G. (1986). DNA content and prognosis in renal cell carcinoma. A comparision between primary tumors and metasates. *Cancer*, **57**, 2346–2350.

LJUNGBERG B, STENLING R AND ROOS G. (1988). Tumor spread and DNA content in human renal cell carcinoma. *Cancer Res.*, **48**, 3165–3167.

LJUNGBERG B, LARSSON P, ROOS G, STENLING R AND WILSON G. (1994). Cell kinetics of renal cell carcinoma studied with in vivo iododeoxyuridine incorporation and flow cytometry. *J. Urol.*, **151**, 1509–1513.

MASTERS JRW, CAMPLEJOHN RS, CONSTANCE PARKINSSON M, WOODHOUSE CRJ AND O'REILLY SM. (1992). Does DNA flow cytometry give useful prognostic information in renal parenchymal adenocarcinoma? *Br. J. Urol.*, **70**, 364–369.

MEHLE C, LJUNGBERG B, STENLING R AND ROOS G. (1993). DNA fingerprinting of renal cell carcinoma with special reference to tumor heterogeneity. *Genes, Chrom. Cancer*, **6**, 86–91.

MEHLE C, LJUNGBERG B AND ROOS G. (1994). Telomere shortening in renal cell carcinoma. *Cancer Res.*, **54**, 236–241.

NICOLSON GL. (1987). Tumor cell instability, diversification, and progression to the metastatic phenotype: from oncogene to oncofetal expression. *Cancer Res.*, **47**, 1473–1487.

NORDENSON I, LJUNGBERG B AND ROOS G. (1988) Chromosomes in renal carcinoma with reference to intratumor heterogeneity. *Cancer Genet. Cytogenet.*, **32**, 35–41.

NORMING U, TRIBUKAIT B, GUSTAFSON H, NYMAN CR, WANG N AND WIJKSTRÖM H. (1992). Deoxyribonucleic acid profile and tumor progression in primary carcinoma in situ of the bladder: a study of 63 patients with grade 3 lesions. *J. Urol.*, **147**, 11-15.

NOWELL PC. (1986). Mechanisms of tumor progression. *Cancer Res.*, **46**, 2203-2207.

ROBSON C, CHURCHILL B AND ANDERSON W. (1969). The results of radical nephrectomy for renal cell carcinoma. *J. Urol.*, **101**, 297-301.

SASAKI K, MURAKAMI T, KAWACHINO K AND TAKAHASHI M. (1988). Intratumoral regional differences in DNA ploidy of gastrointestinal carcinomas. *Cancer*, **62**, 2569-2575.

SASAKI K, MURAKAMI T, MURAKAMI T, NAKAMURA M. (1991). Intratumoral heterogeneity in DNA ploidy of eosophageal squamous cell carcinomas. *Cancer*, **68**, 2403-2406.

SKINNER D, COLVIN R, VERMILLION D, PFISTER RC AND LEADBETTER W. (1971). Diagnosis and management of renal cell carcinoma. A clinical and pathological study of 309 cases. *Cancer*, **28**, 1165-1177.

VOGELSTEIN B, FEARON ER, KERN SE, HAMILTON SR, PREISINGER AC, NAKAMURA Y AND WHITE R. (1989). Allelotype of colorectal carcinomas. *Science*, **244**, 207-211.

WEISS L. (1985). *Principles of Metastasis*. Academic Press: Orlando.